# A Novel Pedestrian Navigation Algorithm for a Foot-Mounted Inertial-Sensor-Based System

**DOI:** 10.3390/s16010139

**Published:** 2016-01-21

**Authors:** Mingrong Ren, Kai Pan, Yanhong Liu, Hongyu Guo, Xiaodong Zhang, Pu Wang

**Affiliations:** 1 School of Electronic Information & Control Engineering, Beijing University of Technology, Beijing 100124, China; KaiPan_com@163.com (K.P.); liu1253891321@163.com (Y.L.); 18810353933@163.com (H.G.); 18811576642@163.com (X.Z.); wangpu@bjut.edu.cn (P.W.); 2 Engineering Research Center of Digital Community, Ministry of Education, Beijing 100124, China; 3 Beijing Key Laboratory of Computational Intelligence and Intelligent Systems, Beijing 100124, China; 4 Beijing Laboratory for Urban Mass Transit, Beijing 100124, China

**Keywords:** Kalman filter, foot-mounted PNS, ZUPT

## Abstract

This paper proposes a novel zero velocity update (ZUPT) method for a foot-mounted pedestrian navigation system (PNS). First, the error model of the PNS is developed and a Kalman filter is built based on the error model. Second, a novel zero velocity detection algorithm based on the variations in speed over a gait cycle is proposed. A finite state machine including three states is employed to model a gait cycle. The state transition conditions are determined based on speed using a sliding window. Third, the ZUPT software flow is illustrated and described. Finally, the performances of the proposed method and other methods are examined and compared experimentally. The experimental results show that the mean relative accuracy of the proposed method is 0.89% under various motion modes.

## 1. Introduction

The rapid development of micro-electromechanical systems (MEMS) has facilitated the production of inexpensive, lightweight and small-sized inertial sensors with low power consumption. These properties are desirable for a PNS. PNS’ that employ MEMS inertial sensors are proposed in [1,2,3,4,5,6]. In this paper, a strapdown inertial navigation algorithm [7,8] is applied to a PNS. Such a PNS is useful for locating and guiding emergency first responders, blind individuals and security personnel.

The major challenge of MEMS-based PNS concerns how to restrain the rapid accumulation of navigation errors. For example, position error is proportional to time cubed. Without an error-resetting algorithm, this error can exceed a meter in ten seconds [1]. A zero velocity update (ZUPT) algorithm could be employed if the PNS is mounted on a shoe. During normal walking cycles, a foot periodically returns to a stationary state and remains on the ground for a brief period of time (approximately 0.1~0.3 s); this interval is referred to as the zero velocity interval. When a stationary state is detected, the velocity error can be employed as an observation to estimate and can correct the sensor bias errors, attitude errors and position errors using a Kalman filter [9,10]. Researches show that the performance of a PNS can be significantly increased using a ZUPT algorithm. 

The detection of the above static periods is a critical step in ZUPT, and many detection methods have been developed [5,9,10,11]. In [11], the zero velocity intervals are determined based on a likelihood ratio test (LRT) detector. The detector provides good performance at low gait speeds (approximately 0.83 m/s). In [5], an algorithm based on a hidden Markov model is constructed using a segmentation of gyroscope outputs. The algorithm shows good reliability under walking and running conditions. However, the state transition model is complex and not easy to implement. In [9], a stance phase detector that consists of one footstep detector and two zero velocity detectors is proposed. The detector can successfully detect zero velocity during walking, stair climbing, and running. However, the detector is easily confused when the pedestrian randomly alternates between walking and running. In this paper, a zero velocity detection algorithm based on the variations in speed during a gait cycle is proposed.

This paper is organized into seven sections. In Section 2, the inertial navigation algorithm of the PNS is provided. In Section 3, the dynamic and measurement models for the ZUPT Kalman filter are summarized. In Section 4, foot-stance phase detection based on variations in speed is presented. In Section 5, the software flow of the PNS is provided. The performance of the proposed method is experimentally verified in Section 6. The last section includes the conclusion and directions for future studies.

## 2. Inertial Navigation Algorithm of the PNS

The inertial navigation algorithm of a PNS is similar to traditional inertial navigation algorithm [12,13]; however, certain simplifications are made based on the characteristics of a PNS.

The body frame is defined as a right-handed (*x*,*y*,*z*) Cartesian coordinate system (*x*—forward, *y*—left, *z*—upward). The navigation frame is defined as east-north-up (ENU). We use the subscripts *b* (body) and *n* (navigation) to denote the project of a vector in a corresponding frame.

The traditional complete form of the velocity differential is:

(1) $${\dot{V}}^{n}=C_{b}^{n}f_{ib}^{b}-(2\omega_{ie}^{n}+\omega_{en}^{n})\times V^{n}+g^{n}$$

where $C_{b}^{n}$ is the transformation matrix from the b frame to the n frame and $f_{ib}^{b}$ is the specific force in the body frame. 

For a PNS, the term $(2\omega_{ie}^{n}+\omega_{en}^{n})\times V^{n}$ can be omitted, and $g^{n}$ can be regarded as approximately constant.

The differential equation of the attitude is:

(2) $${\dot{C}}_{b}^{n}=C_{b}^{n}(\omega_{nb}^{b}\times )$$

$\omega_{nb}^{b}$ represents the angular rate of the body frame relative to the navigation frame. $\omega_{nb}^{b}\times$ represents the cross product matrix of $\omega_{nb}^{b}$.

The position can be updated using the following difference equation:

(3) $$R^{n}(k)=R^{n}(k-1)+V^{n}(k)T_{s}$$

where *T_s_* is the sampling period and is set to 10 m·s. The calculation period of the attitude, velocity and position is equal to *T_s_*.

## 3. ZUPT Kalman Filter

### 3.1. Error Model of Attitude

Let Ψ be the rotation vector from the navigation frame n to the computed navigation frame $n^{\prime }$; then:

(4) $$\dot{\Psi }=-\varepsilon^{n}=-C_{b}^{n}\varepsilon^{b}=-C_{b}^{n}(\varepsilon_{r}^{b}+\varepsilon_{g}^{b})$$

where ε is the vector of the gyroscope measurement error, $\varepsilon_{r}$ is white noise, and $\varepsilon_{g}$ is the gyro bias. The gyro bias is regarded as a first-order Markov process, ${\dot{\varepsilon }}_{g}=-\frac{1}{T_{g}}\varepsilon_{g}+\omega_{g}$, where *T_g_* is the correlation time, and ***ω****_g_* is the driven noise with covariance $\sigma_{g}^{2}$.

### 3.2. Error Model of Velocity

The error model of velocity is given by:

(5) $$\delta {\dot{V}}^{n}=f^{n}\times \psi +\nabla^{n}=C_{b}^{n}f^{b}\times \psi +C_{b}^{n}\nabla^{b}$$

where $\nabla^{b}=\nabla_{r}^{b}+\nabla_{a}^{b}$ is the vector of the accelerometer measurement error, $\nabla_{r}$ is white noise, and $\nabla_{a}$ is the accelerometer bias. The accelerometer bias is modeled as a first-order Markov process, namely, ${\dot{\nabla }}_{a}=-\frac{1}{T_{a}}\nabla_{a}+\omega_{a}$ where $T_{a}$ is the correlation time and $\omega_{a}$ is the driven noise with covariance $\sigma_{a}^{2}$.

### 3.3. Error Model of Position

The error model of position is expressed as:

(6) $$\delta {\dot{R}}^{n}=\delta V^{n}$$

### 3.4. Kalman Filter Equations

The state vector that is employed in the KF has the form: $x=(\Psi^{b},\delta v^{n},\varepsilon_{g}^{b},\nabla_{a}^{b})$ where $\Psi^{b}$ represents attitude errors, $\delta v^{n}$ is the velocity errors, $\varepsilon_{g}^{b}$ is the gyro bias., and $\nabla_{a}^{b}$ is the accelerometer bias.

The dynamic error model of the Kalman filter is given by Equation (7):

(7) $$\dot{x}=\left[\begin{array}{cccc} 0 & 0 & -c_{b}^{n}I & 0\\ f^{n}\times & 0 & 0 & c_{b}^{n}\\ 0 & 0 & -\frac{1}{T_{g}}I & 0\\ 0 & 0 & 0 & -\frac{1}{T_{a}}I \end{array}\right]x+\left[\begin{array}{c} \varepsilon_{r}\\ \nabla_{r}\\ \omega_{g}\\ \omega_{a} \end{array}\right]$$

The measurement equation can be modeled as:

(8) Z=Hx+v

(9) $$H=[{\begin{array}{ccc} 0_{3\times 3} & I_{3\times 3} & 0 \end{array}}_{6\times 3}]$$

The observation δv is the difference between the INS velocity and zero, v is the measurement noise and $E[v(t_{1})v(t_{2})]=Q_{v}\delta (t_{1}-t_{2})$. When the ZUPT is applied, the pedestrian’s foot is usually not perfectly stationary. The uncertainty is modeled in the measurement covariance matrix. ***Q_v_*** is set to (0.05 m/s)^2^*I*_3×3_ based on experimental results.

### 3.5. Feedback Compensation

The estimated state values are used as feedback to the navigation algorithm to correct the corresponding errors. Observation analysis indicates that the rank of the observation matrix $T_{obser}$ is nine, which is less than twelve. Additional analyses conclude that heading error and gyroscope bias errors cannot be observed. Thus, heading error and $\varepsilon_{g}^{b}$ are not used as feedback for the filter. 

The position errors are corrected using Equation (10):

(10) $$\delta R(t)=\frac{1}{2}\delta V^{n}(t)t$$

## 4. Zero Velocity Detection

### 4.1. Variations in Velocity in a Gait Cycle

**Figure 1:**
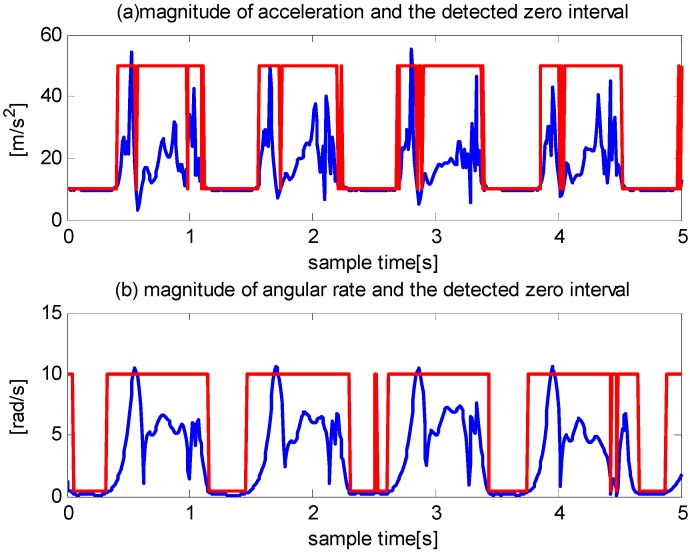
The detected zero velocity interval when a pedestrian is walking. The red line represents the stationary state and moving state. Small values indicate the stationary state. Large values indicate the moving state.

**Figure 2:**
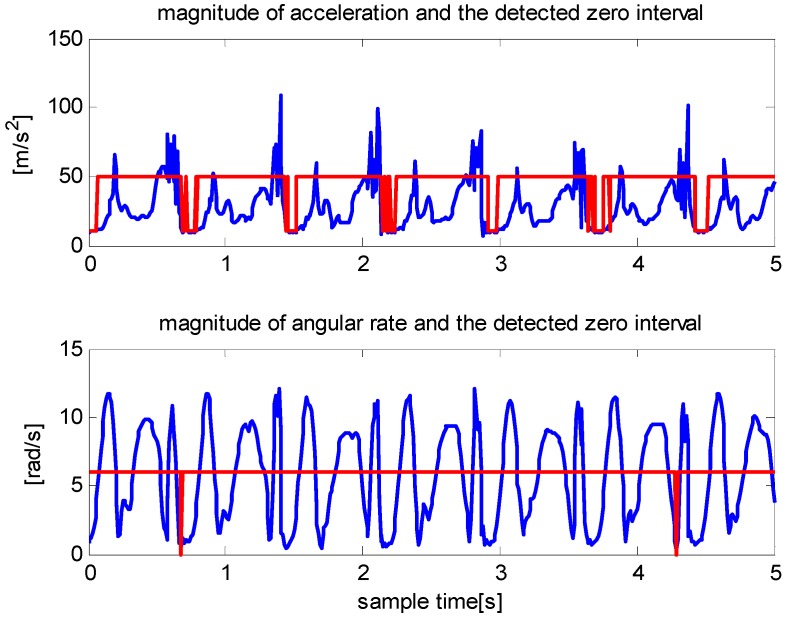
The detected zero velocity interval when a pedestrian is running. In these time intervals, there are no false detections for the accelerometer (**upper**). However, only two zero velocity intervals are detected for the gyroscope (**lower**).

Most zero velocity detection methods employ comparisons between thresholds and the magnitude of acceleration, magnitude of angular rate, or their combinations. The primary constraint of these methods is that the variations in acceleration and angular rate differ greatly under various motion modes, such as walking, running, and stair climbing. Thus, it is difficult to find a threshold function or threshold value that is widely applicable. We demonstrate this in Figure 1 and Figure 2.

Two traditional methods are employed: comparisons of acceleration magnitudes with a threshold and comparisons of angular rate magnitudes with a threshold. Figure 1 shows that although the angular rate magnitude detector performs better than the acceleration-magnitude detector, both methods produce false positives during walking. Figure 2 shows that the acceleration magnitude detector performs better than the angular rate magnitude detector during running; the latter failed to detect most of the zero velocity intervals.

**Figure 3:**
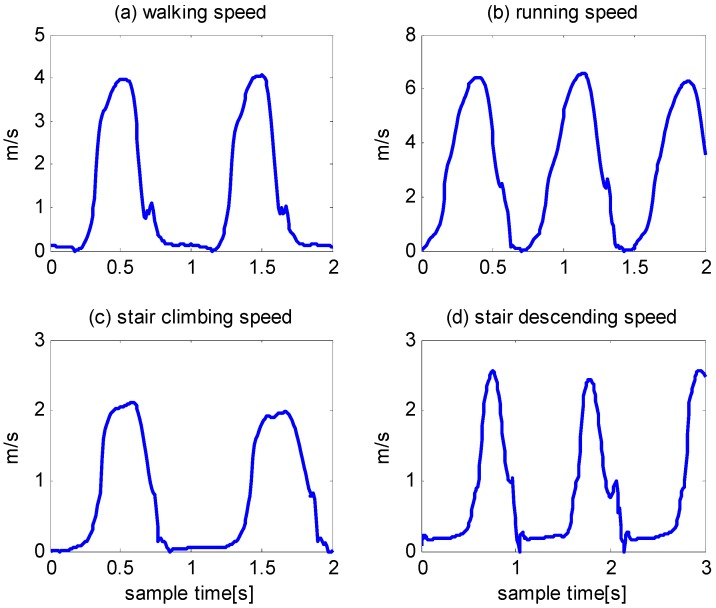
Norm of velocity for different types of gait.

As shown in Equations (1) and (2), the calculation of the velocity uses both acceleration and angular rate data; thus, we attempt to use speed (the norm of velocity) to detect zero velocity intervals. Figure 3 shows the speeds that correspond to walking, running, stair climbing and stair descending. The variations in speed during a gait cycle are similar under different motion modes, except magnitude, duration and certain local details. A comparison of Figure 3 with Figure 1 and Figure 2 show that the variation in speed is simpler and more distinct than the variations in acceleration and angular rate.

**Figure 4:**
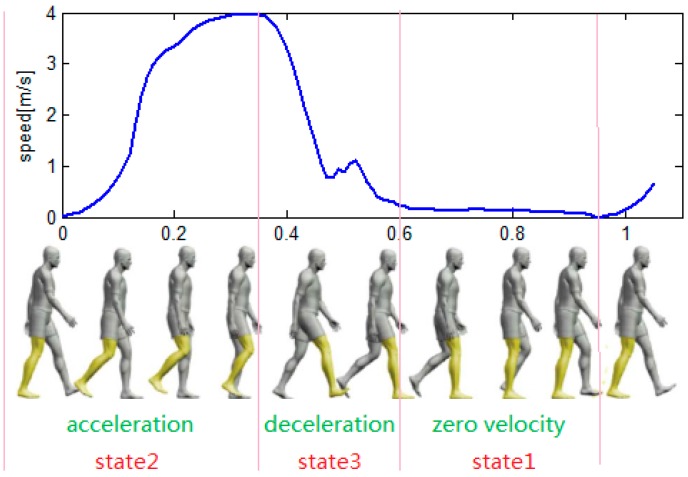
Speed trend and human limb kinematic in a typical walking cycle.

Figure 4 shows the speed trend and a human limb kinematic during a typical walking cycle. The lower panel is taken from [14]. During a walking cycle, the speed changes from acceleration to deceleration, and then changes back to zero. The acceleration interval corresponds to the change from the heel lift to the swing at the highest point. The deceleration interval corresponds to the change from the highest point to a flat foot. The zero velocity intervals correspond to the change from a flat foot to a heel lift.

### 4.2. Hidden Markov Model for Zero Velocity Detection

**Figure 5:**
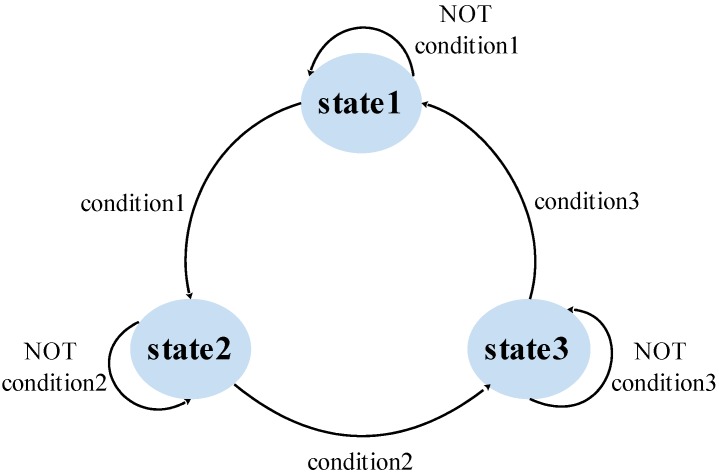
State transition diagram.

We introduce a hidden Markov model for zero velocity detection. Gait cycles are modeled as a finite state machine and each gait cycle is divided into three states. The state transition diagram is shown in Figure 5. State 1 corresponds to zero velocity intervals. State 2 corresponds to acceleration intervals. State 3 corresponds to deceleration intervals.

A sliding window is used to determine state transitions. The size of the sliding window is $N\times T_{s}$. The speeds of the N+1 moments, $t_{k},t_{k+1}\cdots t_{k+N+1}$, are saved in a buffer. If the current state is state 1 and the slope of the speed in the window, denoted by slope_s, is larger than the threshold *Th_a* (condition 1), then the state moves to state 2. If the current state is state 2 and the speed of $t_{k}$ is the maximum speed in the window (condition 2), the state moves to state 3. If the current state is state 3 and the speed of *t_k_* is the minimum speed in the window (condition 3), the state moves to state 1. *Th_a* is set to 0.3 m/s^2^. N is set to 20.

The first order polynomial fitting method is employed to calculate the slope of the speed, as shown in Equation (11):

(11) $$w\_s(i)=s_{0}+s_{1}\times (i-1)\times T+z_{i},i=1,2,\cdots N+1$$

*slope_s*=*s*_1_ is the slope of the speed, which can be interpreted as the mean acceleration in the window.

## 5. PNS Software Flow

**Figure 6:**
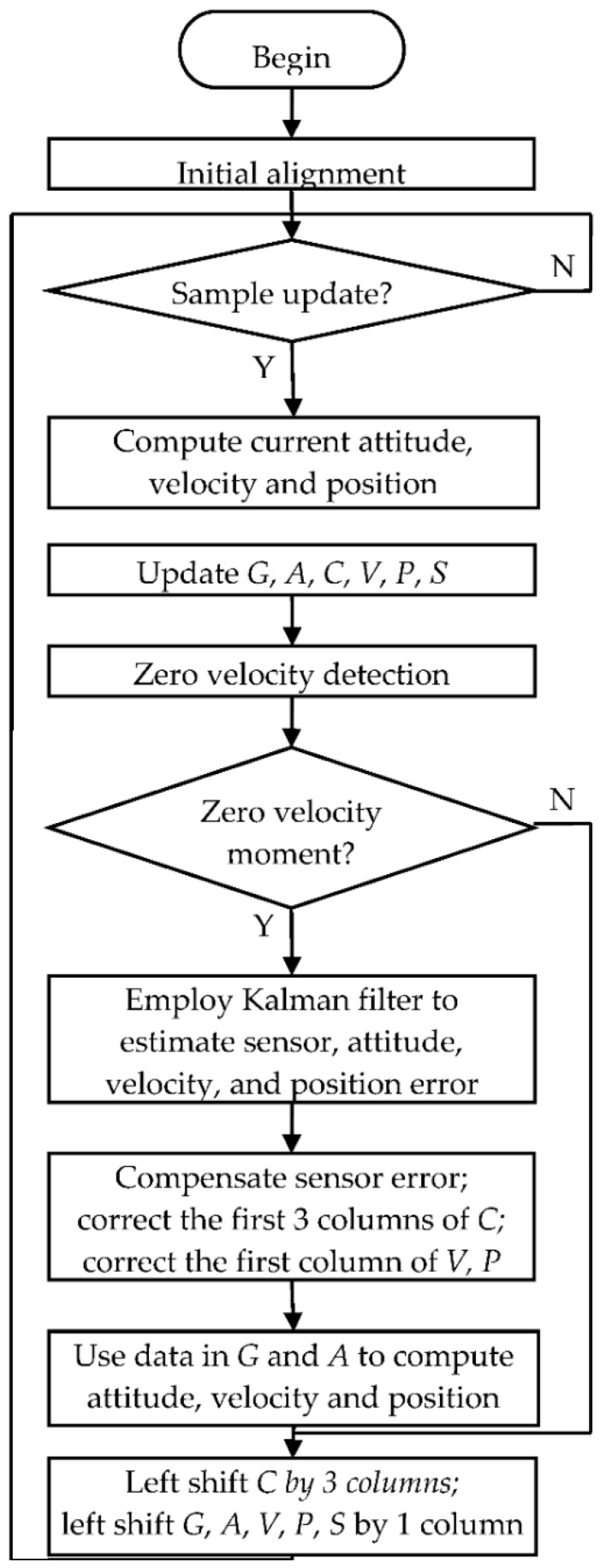
Pedestrian navigation system software flow chart.

The flow chart for the PNS software algorithm is shown in Figure 6.

During the initial alignment, the PNS is initialized with the attitude and position. The initial pitch and roll are calculated using accelerometer data [15], The initial azimuth is calculated using magnetometer data [15], the outdoor position is initialized using GPS data, and the indoor position is initialized using the building plan data, as shown in [2].

The bias of the gyroscope is estimated during initial alignment as well, too. The average of the gyroscope data is calculated and used as the approximate gyroscope bias. If the variance of the gyroscope data is greater than a preset threshold, or if the zero velocity detector has detected motion of pedestrian in less than 6 s, the estimation of the gyroscope bias is skipped.

The matrices ***G***_3×*N*_ and ***A***_3×*N*_ are employed to store the sampling data of the gyroscopes and accelerometers in the window. The matrices ***C***_3×3(*N*+1)_, ***V***_3×(*N*+1)_, ***P***_3×(*N*+1)_ and ***S***_1×(*N*+1)_ are employed to store the attitude matrix, velocity, position and speed in the window.

The zero velocity detection algorithm decides whether the velocity at the starting moment of the window is zero. If the zero velocity moment is detected, attitude errors, velocity errors, and position errors are estimated by the Kalman filter and compensated; then, the sampling data in ***G***_3×*N*_ and ***A***_3×*N*_ are used to recalculate the attitude, velocity and position in the window. After each navigation calculation and ZUPT calculation, ***C***_3×3(*N*+1)_ is left shifted by three columns, and ***G***_3×*N*_, ***A***_3×*N*_, ***V***_3×(*N*+1)_, ***P***_3×(*N*+1)_ and ***S***_1×(*N*+1)_ are left shifted by one column.

## 6. Experiments

### 6.1. The Inertial Measurement Unit

**Figure 7:**
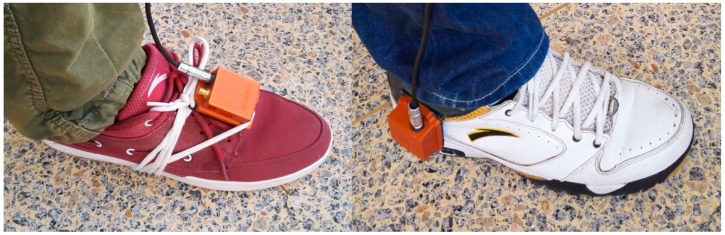
(**Left**) IMU strapped on the front of a shoe; (**Right**) IMU strapped on the rear side of a shoe.

The inertial measurement unit that we employed is the model MTI-G from Xsens Technologies B.V. (Enschede, The Nethelands). It is configured to provide inertial data at 100 Hz. The IMU includes three MEMS accelerometers, three MEMS gyroscopes and three magnetometers. The gyroscope bias repeatability is 0.5 deg/s (max), and the in-run bias stability is 10 deg/h (typical). The accelerometer bias repeatability is 0.05 m/s^2^ (max),and the in-run bias stability is 40 μg (typical). The IMU is strapped on to the front of a shoe or the rear part side of a shoe, as shown in Figure 7.

### 6.2. Zero Velocity Detection

**Figure 8:**
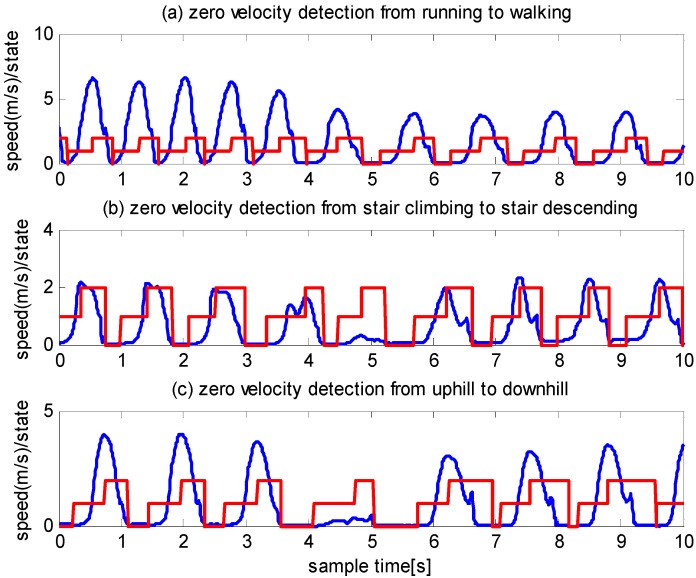
Zero velocity detection using the method proposed in this paper.

The experiments were conducted using a pedestrian, a 25-year-old male with a height of 1.73 m and a weight of 70 kg. The IMU was strapped on to the front of a shoe. The pedestrian conducted six types of motion: walking, running, stair climbing, stair descending, uphill and downhill. The average speeds are approximately 1.35, 2.2, 0.7, 1.09, 0.8 and 1.4 m/s, respectively. Figure 8 shows the speeds and the corresponding zero velocity detector states. The blue line represents the speeds, and the red line represents the state. “0” denotes state 1, “1” denotes state 2 and “2” denotes state 3. Figure 8 shows that the zero velocity detector state well matches the variation in speed under all motion modes.

**Figure 9:**
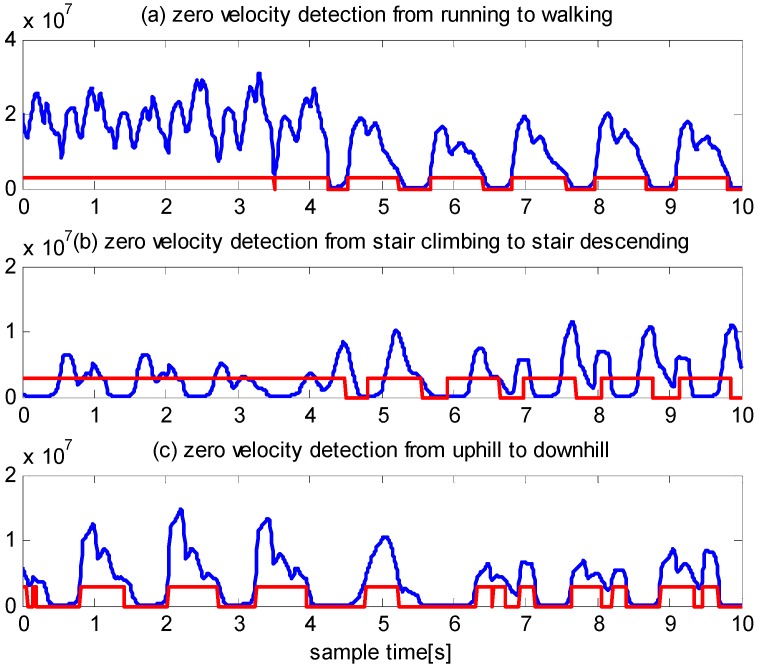
Zero velocity detection using method proposed in [11]. The vertical unite is dimensionless numbers.

To provide a comparison, we examine the performance of the algorithm in [11] using the same experimental data. The detected zero velocity intervals are shown in Figure 9. As illustrated by panel (a), except for one interval, none of the zero velocity intervals, are detected during running. As illustrated by panel (b), none of the zero velocity intervals are detected during stair climbing. Moreover, the detected zero velocity intervals during stair descending are incorrect. As illustrated by panel (c), some of the detected zero velocity intervals during uphill and downhill are correct, although others are incorrect. 

**Figure 10:**
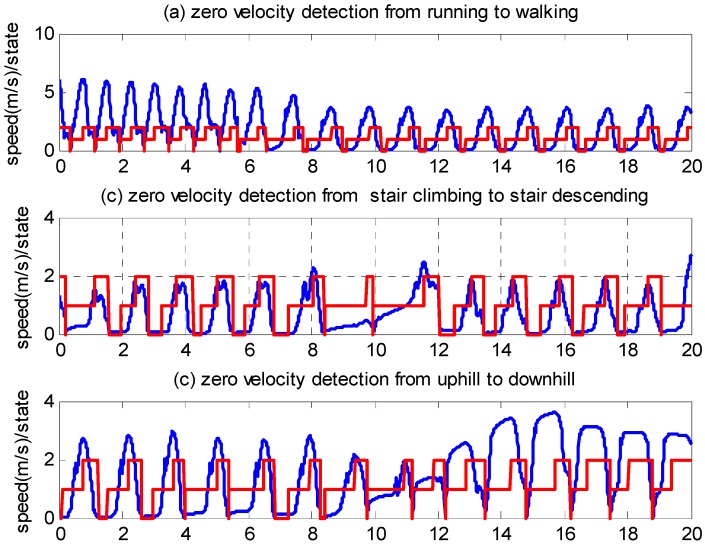
Zero velocity detection of six types of gait when the IMU is strapped onto the rear side of a shoe. The parameters are the same as those in Figure 8.

To examine whether the mounting position of the IMU affects the effectiveness of the proposed algorithm, we conducted experiments with the IMU strapped onto the rear side of a shoe, as illustrated in Figure 7. Six types of motion were performed by a pedestrian, specifically, a 30-year-old male with a height of 1.70 m and a weight of 73 kg. The speed and zero velocity detector states are illustrated in Figure 10, from which it can be observed that the proposed algorithm performed well.

To test the robustness of the algorithm, we randomly selected three other people from the Beijing University of Technology to conduct the experiments. They wore different types of shoes, including leather shoes and sport shoes. One pedestrian conducted the experiments in the same location used by the 25-year-old male. Another two pedestrians conducted the experiment in randomly selected locations in our university. We asked the pedestrians to conduct six motion modes as before. The IMU was strapped onto the front of a shoe. The users can be described as follows: a 26-year-old female with a height of 1.58 m and a weight of 49 kg, a 40-year-old male with a height of 1.78 m and a weight of 62 kg, and a 38-year-old female with a height of 1.60 m and a weight of 51 kg. We carefully compare the performance of the proposed algorithm and the algorithm of [11] and draw the following conclusion:

(1) The algorithm presented in [11] performs well during slow walking but does not perform well during running, stair climbing and descending, uphill and downhill.
(2) The algorithm proposed in this paper performs well under all the above-mentioned types of motion.

### 6.3. Field Test Experiments

**Figure 11:**
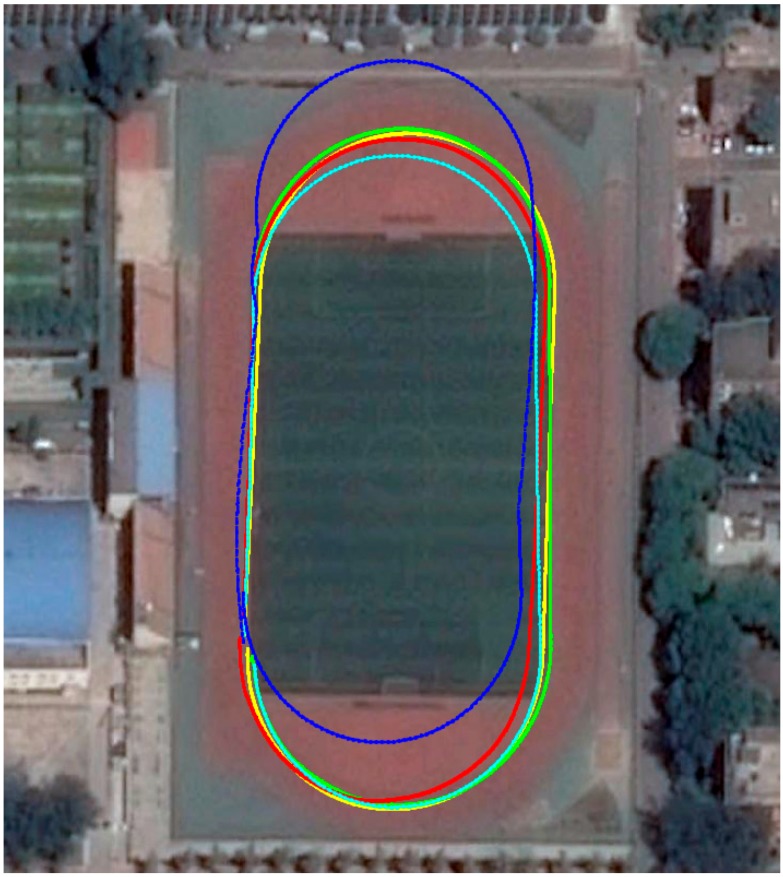
Comparison of the calculated trajectories between the proposed algorithm and algorithm [11].

Experiments under three motion modes, including walking, running and alternating between walking and running, were conducted around the boundary line of the sports field at the Beijing University of Technology. The travelled distance is approximately 422 m. The calculated trajectories of the proposed algorithm and of that in [11] are shown in Figure 11. The IMU was strapped onto the front of a shoe. The blue line (walking alternating with running) and light blue (walking) line are the calculated trajectories of algorithm [11]. The red line (walking), yellow line (walking alternating with running) and green line (running) are calculated trajectories of the proposed method. The calculated trajectory of method [11] under the running mode is not illustrated because of the excessive position errors. The mean error (start/end distance) of the proposed algorithm is approximately 4.64 m, or approximately 1.10% of the travelled distance. The mean error (start/end distance) of the algorithm [11] is approximately 15.82 m, or approximately 3.75% of the travelled distance.

**Figure 12:**
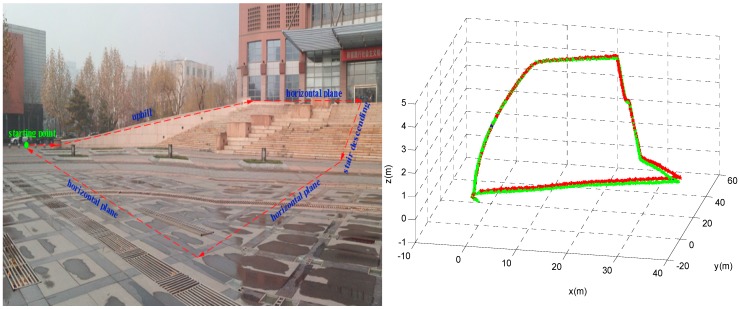
Uphill and stair descending trajectory. Path used for testing (**left**). The calculated trajectories of the proposed algorithm (**right**).

We also conducted experiments along another more complex trajectory that included uphill movement and stair descending as illustrated by the red dashed line in Figure 12. The pedestrian walked along the trajectory defined by the red dashed line. She stopped for a few seconds when moving uphill (blue), on the horizontal plane (red) and when descending the stair (green). The travelled distance is approximately 147 m, and the mean error is approximately 0.68% of the travelled distance.

Table 1 summarizes the final return position errors along the two trajectories. The maximum error is 1.61% during running along trajectory 1. The mean error of the two trajectories is 0.89%. The data in Table 1 indicate that the proposed algorithm provided robust positioning results under various motion modes and with different pedestrians.

**Table 1:** Field test return position errors.

Event		Average Speed (m/s)	Travelled Distance (m)	Position Error (%)
Trajectory 1	Walking	1.3	422	0.60%
	Running	2.6	421	1.61%
	Running and Walking	1.8	422	1.10%
Trajectory 2	Stop on the uphill	0.8	148	0.44%
	Stop on the horizontal plane	0.7	147	0.67%
	Stop on the stair descending	1.2	147	0.93%

## 7. Conclusions

The experimental results show that the algorithm proposed in this paper can correctly detect zero velocity intervals under various motion modes. The algorithm is insensitive to the mounting position of the IMU and differences in pedestrians.

We also conducted experiments under an elevator environment. Because the acceleration of the elevator was lower than the threshold *Th_a*, the ZUPT state machine remained in the zero velocity state regardless of whether the elevator was rising or descending. We will attempt to solve this problem in the future.

ZUPT cannot estimate or compensate for heading errors, which are a primary error source in PNS. Other techniques, such as received signal strength (RSS) measurements [16], map matching [2,17], building heading [7] or computer vision-derived position measurements [18] can be employed to improve the accuracy of a PNS. We will discuss the integration of these techniques with ZUPT in a future paper.

## References

[1] Foxlin E. (2005). Pedestrian Tracking with Shoe-Mounted Inertial Sensors. IEEE Comput. Graph. Appl..

[2] Pinchin J., Hide C., Moore T. A Particle Filter Approach to Indoor Navigation Using a Foot Mounted Inertial Navigation System and Heuristic Heading Information. Proceedings of the 2012 International Conference on Indoor Positioning and Indoor Navigation (IPIN).

[3] Zhang R., Hoflinger F., Reindl L. (2013). Inertial Sensor Based Indoor Localization and Monitoring System for Emergency Responders. IEEE Sens. J..

[4] Bird J., Arden D. (2011). Indoor navigation with foot-mounted strapdown inertial navigation and magnetic sensors [Emerging Opportunities for Localization and Tracking]. IEEE Wirel. Commun..

[5] Park S.K., Suh Y.S. (2010). A zero velocity detection algorithm using inertial sensors for pedestrian navigation systems. Sensors.

[6] Olivares A., Ramirez J., Gorriz J.M., Olivares G., Damas M. (2012). Detection of (in)activity periods in human body motion using inertial sensors: A comparative study. Sensors.

[7] Abdulrahim K., Hide C., Moore T., Hill C. (2011). Aiding Low Cost Inertial Navigation with Building Heading for Pedestrian Navigation. J. Navig..

[8] Sabatini A.M. (2011). Estimating three-dimensional orientation of human body parts by inertial/magnetic sensing. Sensors.

[9] Li Y., Wang J.J. A robust pedestrian navigation algorithm with low cost IMU. Proceedings of the 2012 International Conference on Indoor Positioning and Indoor Navigation (IPIN).

[10] Romanovas M., Goridko V., Al-Jawad A., Schwaab M., Traechtler M., Klingbeil L., Manoli Y. A Study on Indoor Pedestrian Localization Algorithms with Foot-Mounted Sensors. Proceedings of the 2012 International Conference on Indoor Positioning and Indoor Navigation (IPIN).

[11] Skog I., Handel P., Nilsson J.O., Rantakokko J. (2010). Zero-velocity detection—An algorithm evaluation. IEEE Trans. Bio-Med. Eng..

[12] Titterton D.H., Weston J.L. (2004). Strapdown Inertial Navigation Technology.

[13] Qin Y.Y. (2014). Inertial Navigation.

[14] Kwakkel S.P. GNSS Aided *In Situ* Human Lower Limb Kinematics During Running. Proceedings of the International Technical Meeting of the Satellite Division of the Institute of Navigation.

[15] Huang C., Liao Z., Zhao L. (2010). Synergism of INS and PDR in Self-Contained Pedestrian Tracking with a Miniature Sensor Module. IEEE Sens. J..

[16] Ruiz A.R.J., Granja F.S., Honorato J.C.P., Rosas J.I.G. Pedestrian Indoor Navigation by aiding a Foot-mounted IMU with RFID Signal Strength Measurements. Proceedings of the 2010 International Conference on Indoor Positioning and Indoor Navigation.

[17] Zampella F., Jimenez Ruiz A.R., Seco Granja F. (2015). Indoor Positioning Using Efficient Map Matching, RSS Measurements, and an Improved Motion Model. IEEE Trans. Veh. Technol..

[18] Placer M., Kovacic S. (2013). Enhancing indoor inertial pedestrian navigation using a shoe-worn marker. Sensors.

